# Piloting work of outreach nurses in Harm Reduction projects in Ukraine

**DOI:** 10.1186/1742-4690-9-S1-P117

**Published:** 2012-05-25

**Authors:** Natalya Dvinskykh, Sergiy Botvin

**Affiliations:** 1 HIV AIDS Alliance in Ukraine, Kyiv, Ukraine

## 

In Ukraine, despite growing sexual way of transmission of HIV, drug use remains the driving force of the epidemic. Prevalence of HIV among IDU is the highest compared to other risk groups - 21.6%, according to biobehavioral study conducted in Ukraine in 2009. However IDU often do not have access to most of the needed medical services because of stigma, discriminative attitude, and lack of resources.

To address these issues and to bring basic medical services to the places and at a time convenient for drug isers, a project "outreach nurses was piloted in 2011. The aim of the project was to introduce work of medical professionals (nurses) as a part of Harm Reduction Program. The project was led by All-Ukrainian Harm Reduction Association, supported by HIV/AIDS Alliance in Ukraine and implemented in 5 regions of Ukraine. 3 nurses from each region were trained on two workshops: Harm Reduction Basics and VCT. The nurses provided basic medical services and consultations at mobile clinics, fixed NEPs, outreach routs and clients' houses. They changed bandages, applied ointments, checked body temperature etc., as well as provided information on various health improvement issues and referred to other services.

The intervention proved to be extremely popular among IDU 1732 clients were reached by the services during the project's work. The most popular services included: veins treatment, bandaging, anticeptics dissemination, and medical consultations, including HIV counseling. Those nurses who had not worked in Harm Reduction programs before reported that their attitude changed a lot during the project implementation: from fear to being ready to help and being proud on the ability to help people in need. The new services also gained the most positive feedback from the clients.

The project piloting showed that work of outreach nurses can be useful for any Harm Reduction program and can improve quality of its services. There are several factors that are important for the success of the intervention. Among them: adequate training for outreach nurses, 'nurse - social worker' team work, project's cooperation with local medical institutions.

